# Initial responses of rove and ground beetles (Coleoptera, Staphylinidae, Carabidae) to removal of logging residues following clearcut harvesting in the boreal forest of Quebec, Canada

**DOI:** 10.3897/zookeys.258.4174

**Published:** 2013-01-15

**Authors:** Timothy T. Work, Jan Klimaszewski, Evelyne Thiffault, Caroline Bourdon, David Paré, Yves Bousquet, Lisa Venier, Brian Titus

**Affiliations:** 1 Département des sciences biologiques, Université du Québec à Montréal, CP 8888, succursale Centre-ville, Montréal, Quebec, Canada H3C 3P8; 2 Natural Resources Canada, Canadian Forest Service, Laurentian Forestry Centre, 1055 du P.E.P.S., P.O. Box 10380, Stn. Sainte-Foy, Québec, Quebec, Canada G1V 4C7; 3 Agriculture and Agri-Food Canada, Canadian National Collection of Insects, Arachnids and Nematodes, Ottawa, Ontario, Canada K1A 0C6; 4 Natural Resources Canada, Canadian Forest Service, Great Lakes Forestry Centre, 1219 Queen Street East, Sault Ste. Marie, Ontario, Canada P6A 2E5; 5 Natural Resources Canada, Canadian Forest Service, Pacific Forestry Centre, 506 Burnside Road West, Victoria, British Columbia, Canada V8Z 1M5

**Keywords:** Biomass removal, tree harvesting, boreal forest, Coleoptera, Staphylinidae, Carabidae

## Abstract

Increased interest in biomass harvesting for bioenergetic applications has raised questions regarding the potential ecological consequences on forest biodiversity. Here we evaluate the initial changes in the abundance, species richness and community composition of rove (Staphylinidae) and ground beetles (Carabidae), immediately following 1) stem-only harvesting (SOH), in which logging debris (i.e., tree tops and branches) are retained on site, and 2) whole-tree harvesting (WTH), in which stems, tops and branches are removed in mature balsam fir stands in Quebec, Canada. Beetles were collected throughout the summer of 2011, one year following harvesting, using pitfall traps. Overall catch rates were greater in uncut forest (Control) than either stem-only or whole-tree harvested sites. Catch rates in WTH were greater than SOH sites. Uncut stands were characterized primarily by five species: *Atheta capsularis*, *Atheta klagesi*, *Atheta strigosula*, *Tachinus fumipennis*/*frigidus* complex (Staphylinidae) and to a lesser extent to *Pterostichus punctatissimus* (Carabidae). Increased catch rates in WTH sites, where post-harvest biomass was less, were attributable to increased catches of rove beetles *Pseudopsis subulata*, *Quedius labradorensis* and to a lesser extent *Gabrius brevipennis*. We were able to characterize differences in beetle assemblages between harvested and non-harvested plots as well as differences between whole tree (WTH) and stem only (SOH) harvested sites where logging residues had been removed or left following harvest. However, the overall assemblage response was largely a recapitulation of the responses of several abundant species.

## Introduction

Increased interest in the use of forest biomass for bioenergy production has been met with concerns related to potential negative impacts of increased biomass harvesting on biodiversity (Abbas et al. 2011, Berch et al. 2011). Relative to forest harvesting for traditional wood products such as veneer, lumber and pulp, biomass harvesting relies on exploiting a larger diversity of biomass feedstock sources, such as previously non-commercial trees and/or parts of trees. Increased use of a greater variety of biomass feedstock sources including logging residues (i.e. tree tops and branches from harvested trees) will likely reduce potential sources of deadwood and may have direct and indirect ecological repercussions for biodiversity (Stokland 2001).

Litter dwelling beetles have been recognized as useful indicators of forest change and ecosystem functioning (Rainio and Niemelä 2003, Niemelä 2000, Pohl et al. 2007). In some cases, reductions in deadwood have negative effects, particularly on species that are closely associated with deadwood as a developmental substrate for larvae or as a food resource (Siitonen 2001, Martikainen and Kouki 2003, Simila et al. 2003, Jonsson et al. 2005, Work and Hibbert 2011). For certain saproxylic species, reductions in deadwood stemming from forest management, and presumably from biomass harvesting, result in the direct loss of a necessary resource (Jonsell and Weslien 2003, Johansson et al. 2007). For other organisms, such as leaf litter invertebrates, which may possess broader habitat and feeding preferences, evidence for the relation with volumes of woody debris has been limited or not yet well documented (Pearce et al. 2003, Work et al. 2004) or absent (Pearce et al. 2004, Paradis and Work 2011). Reductions in deadwood volume following biomass harvesting could affect leaf litter invertebrates in multiple, non-exclusive ways. Residual deadwood could serve as an important microhabitat that buffers organisms from the increased temperature and reduced humidity that often accompanies the removal of the overstory canopy (Pearce et al. 2003). In this way, deadwood may only become a critical habitat for leaf litter organisms after overstory removal, and would not necessarily be detected in studies where volumes of woody debris between treatments are not drastically different. As many leaf-litter invertebrates are characterized as generalist predators, loss of deadwood caused by biomass harvesting could also signify a loss of potential prey items and possibly truncation of food webs (Komonen et al. 2000). Such responses would likely only be observed through longer-term biomonitoring efforts, whereby any initial reorganization of species assemblages following removal of the overstory could be separated from later community-level effects resulting from debris removal. However, long-term responses of invertebrate assemblages to deadwood will likely be dependent on the initial species filtering that occurs immediately following harvest.

Here we present initial responses of rove and ground beetles to removal of logging residues following clearcut harvesting. As with any study examining a relatively high number of species, we expected to observe a variety of species-specific responses. However as an initial starting hypothesis, we speculated that removal of forest overstory combined with removal of residual forest biomass in the form of logging debris would result in lower abundances of individual species compared with sites where only overstory was removed. We further expected that these would translate to overall assemblage-level differences between harvested sites where additional biomass had been removed and sites that had experienced only clearcut harvesting.

## Methods

### Sampling sites

**Figure 1.**
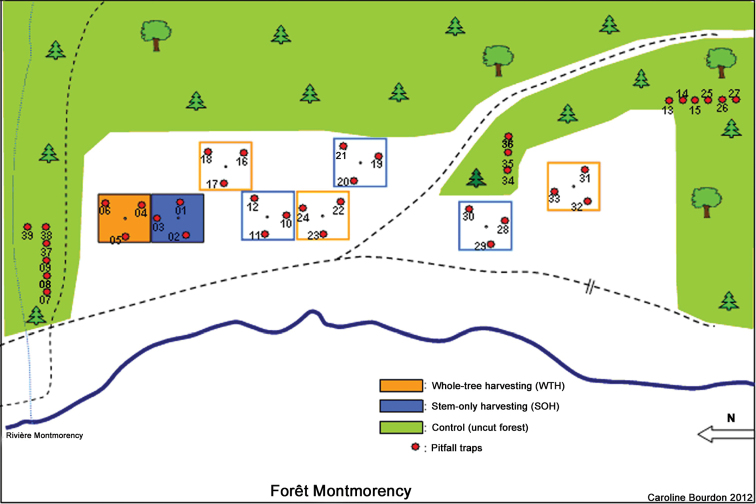
Schematic representation of treatments with pitfall trap locations, Forêt Montmorency, Quebec.

**Figure 2.**
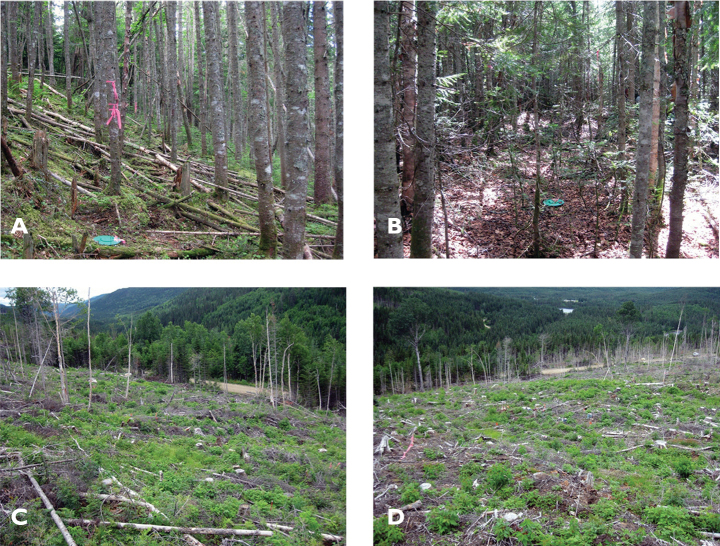
Photographs of experimental plots taken one year following harvest (2012) **A, B** photos of uncut forests (Control) **C** stem-only harvested plot (SOH) (operational level) **D** whole-tree harvested plot (WTH).

We sampled beetles using pitfall traps within the Montmorency Teaching and Research Forest (47°13' and 47°22'N, and 71°05' and 71°11'W)approximately 70 km north of Quebec City, Quebec (Fig. 1). This project is part of a long-term national study on monitoring of the effects of biomass harvesting on forest ecosystem functioning (Thiffault et al. 2011a, Venier at al. 2012). This site is part of a 60-year-old boreal balsam fir/white birch dominated forest in the Laurentian Mountains. The experimental layout was a randomized block design, with four replicates of the following treatments: 1) conventional stem-only harvesting (SOH) (Fig. 2), where all trees with a diameter at breast height (dbh) greater than 9.1 cm were felled, delimbed and topped at the stump and only the stems hauled to the roadside and non-merchantable material such as tops and branches were left distributed evenly throughout the site, 2) whole-tree harvesting (WTH) (Fig. 2), that is, felling of trees with a dbh of 9.1 cm and greater and hauling of stems, tops and branches to the roadside, and 3) uncut forests (Control) (Fig. 2). Treatments were randomly assigned to each experimental plot within a block. Permanent sampling plots were established in each block and consisted of circular plots with a radius of 20 m and situated within 40 × 40 m blocks (Thiffault et al. 2011b). In each circular subplot, three pitfall traps were installed at distances of 10 to 12 m from the centre of the plot, except in two control plots in which six traps were installed in transect configurations spaced at the same distance as in the treatment plots. We used a greater number of traps in control plots to better account for the larger diversity of microhabitats and species in the uncut forest immediately surrounding the experimental plots.

### Estimating woody debris volume

Woody debris volumes were estimated from two 20-m-long perpendicular transects intercepting the centre of each plot using the line intersect method (Thiffault et al. 2011b). Two categories of debris were counted: (1) fine woody debris (FWD), defined as debris from 1.1 to 3 cm in diameter; (2) coarse woody debris (CWD), which includes debris greater than 3 cm in diameter. FWD was counted for the first and the last 5-m section of each transect. The total number of pieces in each 5 m section was tallied. For CWD, the diameter was recorded for each piece along the whole length of the transects.

### Beetle sampling

We used commercially produced pitfall traps 12 cm in diameter (Bio-Control Inc., Quebec City) with rain covers spaced 10-15 m apart (see details of trap design in Klimaszewski et al. 2007). As a killing solution in pitfalls we used 70% ethanol with a few drops of commercially available vinegar to prevent muscle stiffness when mounting specimens. Beetles were collected continuously between June 9 and August 25, 2011. We emptied traps approximately every week during this period. The number of traps was not identical between experimental parcels. In all harvested stands, three pitfall traps were used. For uncut plots, we used three traps in one replicate, and six traps in the remaining two uncut plots. All beetles were sorted by specialist technicians and in the case of rove beetles, mounted and dissected as needed prior to identification. All rove beetles were identified by J. Klimaszewski, and ground beetles by Y. Bousquet. Most aleocharine staphylinid specimens were verified using genitalic characters. Colour images of the most abundant rove and ground beetle species and the lists of all species per family are shown in the Appendix (Figs 7, 8, Tables 4, 5).

### Statistical analysis

Debris volumes for both small and coarse woody material were compared separately among treatments using linear models where the stem-only harvesting (SOH) treatment was chosen as a reference. Abundances were converted to catch rates to account for differences in trapping effort between sites, which varied due to infrequent disturbances to particular traps by vertebrates over the course of the sampling season and the total number of traps placed within each experimental parcel. Abundances at individual traps were pooled to calculate catch rates for each experimental plot. Thus in harvest plots, catch rates reflect the combined trapping effort of three traps over the course of the season (231 trap days/plot). The number of traps per control plot varied between 3 and 6, corresponding to 203 and to 462 total trap days. We compared overall catch rates among silvicultural treatments using simple linear regression where total catch rate was square root transformed to meet assumptions of normality. We also compared catch rates of the 19 most abundant species (those that comprised more than 2% of the total catch rate) among treatments using non-parametric Kruskal-Wallis rank sum tests.

We analyzed differences in beetle species composition using multivariate regression tree (MRT) analysis (De’ath 2002). Multivariate regression trees are used to classify objects (typically sample sites) by maximizing the deviance between splits based on explanatory variables (typically environmental or treatment variables). This method makes a few assumptions regarding the underlying relationship between species and environmental variables and also provides the advantage of visualizing complex interactions among environmental variables (De’ath 2002). For our analysis we used the same matrix of square-root transformed catch rates to create a sum of squares regression tree. We chose a final tree based on a 1000 fold cross-validation procedure. A final tree with two splits was selected 934/1000 times. We used R (2.12.2) for all statistical analysis.

## Results

**Figure 3.**
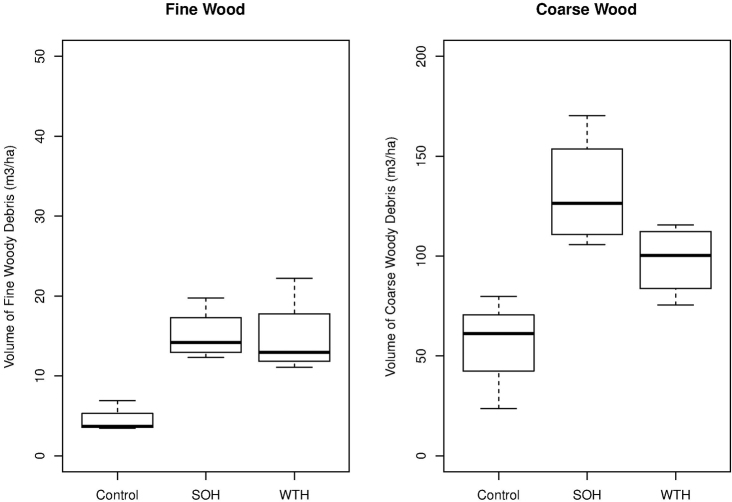
Boxplots showing volume of fine and coarse woody debris in stem-only harvested plots (SOH), whole-tree harvested plots (WTH), and in uncut forest (Control). Bold line depicts median value, box denotes 25–75% quantile range, whiskers correspond to 1.5 times the interquartile range.

Volumes of both fine and coarse debris were higher in clearcuts following harvest as compared with uncut stands (fine debris ANOVA *F_2,8_*=12.73, *P=*0.003, coarse debris ANOVA *F_2,8_*=8.12, *P=*0.011; Fig. 3). Whereas volumes of fine debris were greater in clearcuts, there was no difference in FWD volumes between stem-only and whole-tree harvested plots (*β=-*0.06 (0.35), Wald *t-*value *= -*0.173, *p=* 0.867). For coarse debris (CWD), whole-tree harvested plots had reduced volumes as compared with stem-only harvested plots (*β=*-34.20 (17.74), Wald *t-*value *=* -1.927, *p=* 0.090), although these differences could only be characterized as nearly significant with alpha = 0.05.

**Figure 4.**
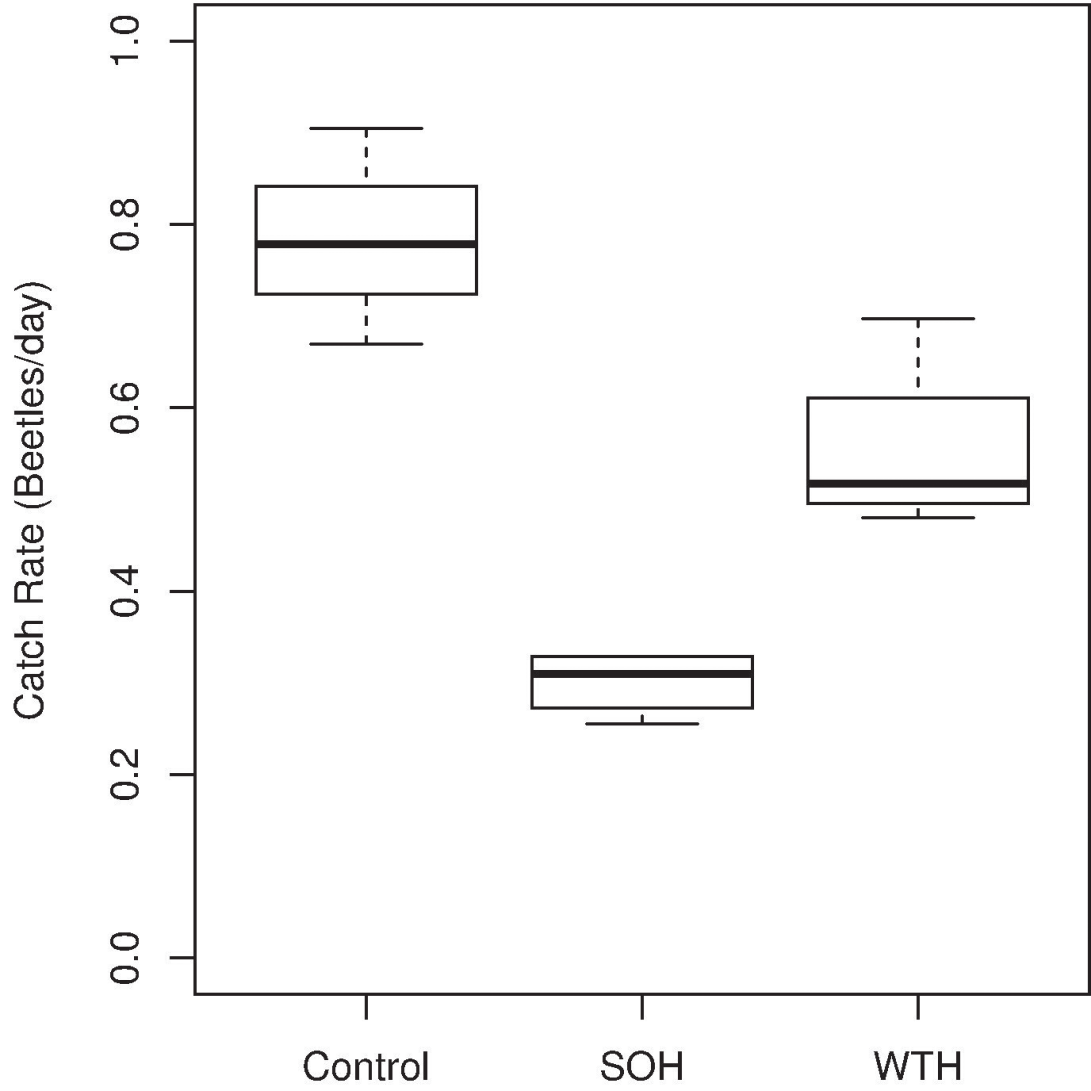
Boxplots depicting overall catch rates (beetles/day) where forest was **a** clearcut and deadwood was left intact (SOH) **b** clearcut with quantity of deadwood reduced (WTH), and **c** uncut forest (Control). Bold line depicts median value, box denotes 25–75% quantile range, whiskers correspond to 1.5 times the interquartile range.

**Figure 5.**
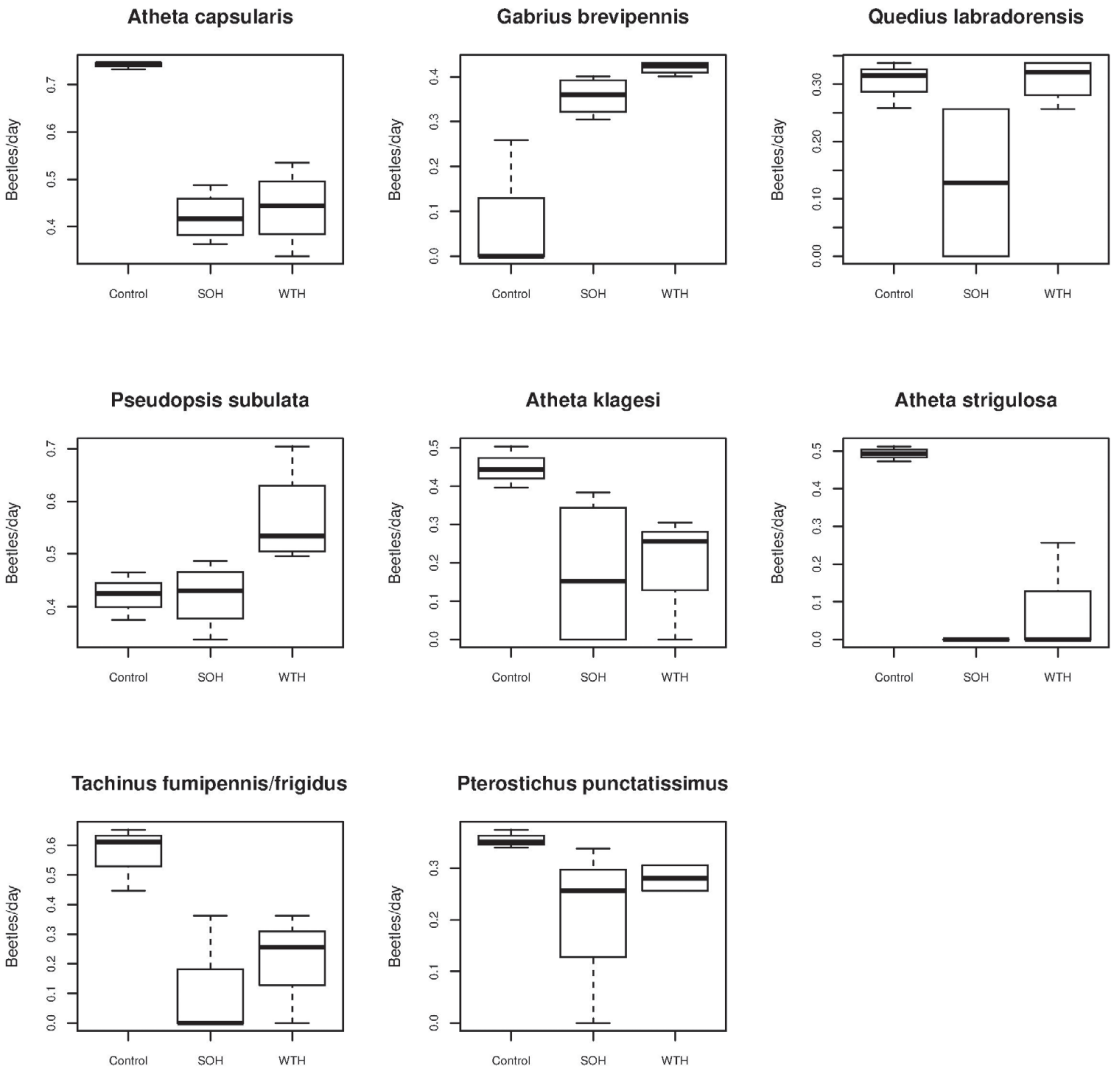
Boxplots depicting catch rates (beetles/day) for eight abundant species collected from experimental plots where forest was **a** clearcut and deadwood was left intact (SOH) **b** clearcut with quantity of deadwood reduced (WTH), and **c** uncut forest (Control). Bold line depicts median value, box denotes 25–75% quantile range, whiskers correspond to 1.5 times the interquartile range.

We collected 70 species of rove and ground beetles representing 1665 individuals between June 9 and August 25, 2011 (Appendix Tables 4 and 5). Of the total number of individuals collected, 1278 (53 species) were rove beetles and 387 (17 species) were ground beetles (Table 1). Harvesting reduced overall catch rates in both SOH sites (*β*=-0.33 (0.041), Wald *t-*value= -8.01, *p*<0.001) and WTH (*β*=-0.14 (0.041), Wald *t-*value= -3.39, *p=*0.009) as compared with control stands (ANOVA *F_2,8_*=33.15, *P<*0.001) (Fig. 4). The high overall abundances in uncut stands is attributable to three species of *Atheta* (*Atheta capsularis*, *Atheta klagesi* and *Atheta strigosula*), *Tachinus fumipennis*/*frigidus* complex and to a lesser extent *Pterostichus punctatissimus* (Table 2, Fig. 5). Increased catch in whole-tree harvested plots was observed for three species: *Pseudopsis subulata*, *Quedius labradorensis* and to a lesser extent *Gabrius brevipennis* (Table 2, Fig. 5). With the exception of these species, we were unable to detect statistically significant differences in catch rates between whole-tree and stem-only harvested plots.

**Table 1.** Abundance of beetle species in dead wood reduced plots (WTH), and in deadwood intact plots (SOH), and in uncut forest (Control). Rove and ground beetle species mixed and listed alphabetically.

**Species**	**WTH**	**SOH**	**Control**
*Acidota quadrata*	0	0	1
*Agonum gratiosum*	0	0	1
*Agonum retractum*	0	0	3
*Aleochara* sp. n.	1	1	0
*Aleochara verna*	0	1	0
*Aleochara fumata*	1	0	0
*Atheta ventricosa*	2	2	15
*Atheta regisalmonis*	0	0	1
*Atheta remulsa*	0	1	1
*Atheta terranovae*	0	0	3
*Atheta (Microdota)* sp.	1	0	1
*Atheta klagesi*	4	7	52
*Atheta capsularis*	40	31	334
*Atheta* sp.	1	0	1
*Atheta strigulosa*	1	0	66
*Atrecus macrocephalus*	10	17	3
*Bembidion grapii*	2	0	0
*Bembidion wingatei*	7	5	6
*Bisnius cephalicus*	1	1	1
*Calathus advena*	0	1	1
*Calathus ingratus*	19	4	12
*Eusphalerum pothos*	0	0	1
*Gabrius brevipennis*	29	16	2
*Gabrius* sp.	0	1	0
*Harpalus laticeps*	0	1	0
*Harpalus rufipes*	4	0	0
*Harpalus solitaris*	3	0	0
*Ischnosoma fimbriatum*	33	25	12
*Ischnosoma pictum*	5	3	1
*Lathrobium washingtoni*	1	2	0
*Leptusa opaca*	1	0	0
*Liogluta aloconotoides*	9	0	11
*Lordithon fungicola*	0	1	0
*Lypoglossa franclemonti*	27	12	1
*Megarthrus* sp.	0	0	1
*Mocyta breviuscula*	0	0	3
*Mocyta fungi*	0	1	0
*Mycetoporus americanus*	5	3	13
*Mycetoporus consors*	0	2	0
*Omalium rivulare*	0	1	0
*Oxypoda frigida*	1	1	1
*Oxypoda grandipennis*	0	1	18
*Oxypoda lacustris*	0	0	3
*Oxypoda operta*	1	0	0
*Oxypoda pseudolustrica*	8	5	2
*Phloeostiba lapponica*	7	2	0
*Placusa incompleta*	0	0	2
*Platynus decentris*	1	2	7
*Proteinus* sp.	2	1	3
*Pseudopsis subulata*	109	32	40
*Pterostichus adstrictus*	91	47	52
*Pterostichus brevicornis*	1	0	1
*Pterostichus coracinus*	24	19	30
*Pterostichus punctatissimus*	6	5	17
*Quedius densiventris*	14	1	5
*Quedius fulvicollis*	4	0	1
*Quedius labradorensis*	9	2	10
*Quedius plagiatus*	0	1	0
*Seeversiella globicollis*	3	2	0
*Sphaeroderus nitidicollis*	0	1	1
*Stenus austini*	2	0	0
*Tachinus fumipennis*	6	4	120
*Tachinus luridus*	0	1	1
*Tachinus quebecensis*	0	0	11
*Tachyporus nitidulus*	9	5	1
*Tachyporus* sp.	1	1	0
*Trechus apicalis*	5	5	1
*Trechus crassiscapus*	0	1	1
*Zyras obliqus*	0	0	1

**Table 2.** Kruskal-Wallis comparison of abundant rove and ground beetle species that responded to harvest.

**Species**	**Kruskal-Wallis Chi-Square**	**df**	**p-value**
**Negatively Affected by Harvesting**			
*Atheta capsularis*	6.13	2	0.047
*Atheta klagesi*	6.18	2	0.046
*Atheta srigulosa*	8.29	2	0.016
*Tachinus fumipennis*	6.78	2	0.034
*Pterostichus punctatissimus*	6.51	2	0.039
**Positively Affected by Harvesting**			
*Pseudopsis subulata*	7.00	2	0.030
*Quedius labradorensis*	6.30	2	0.043
*Gabrius brevipennis*	8.68	2	0.013

**Figure 6.**
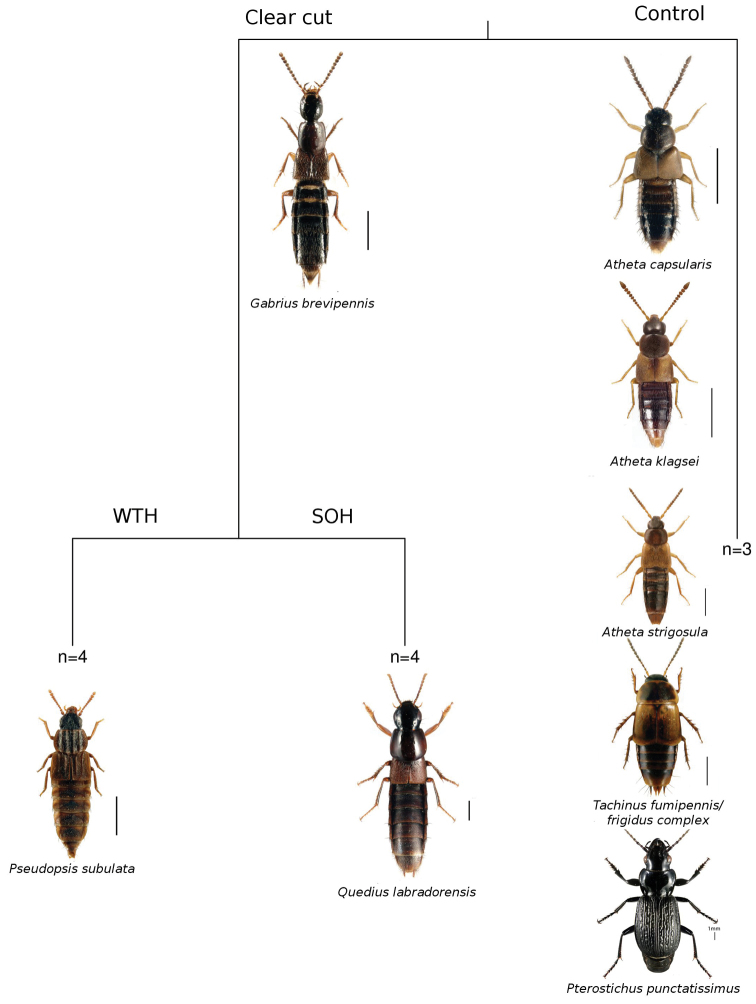
Multivariate regression tree based on sum-of-squares depicting differences in beetle assemblages among experimental plots where forest was **a** clearcut with stem-only harvested (SOH) **b** clearcut with whole-tree harvested (WTH), and **c** uncut forest (Control). The tree was selected based on 935/1000 cross-validations and explains 64% of the variance. Both experimental treatment and deadwood volumes provided equivalent improvement at each split. We have labelled splits using experimental treatments.

**Figure 7.**
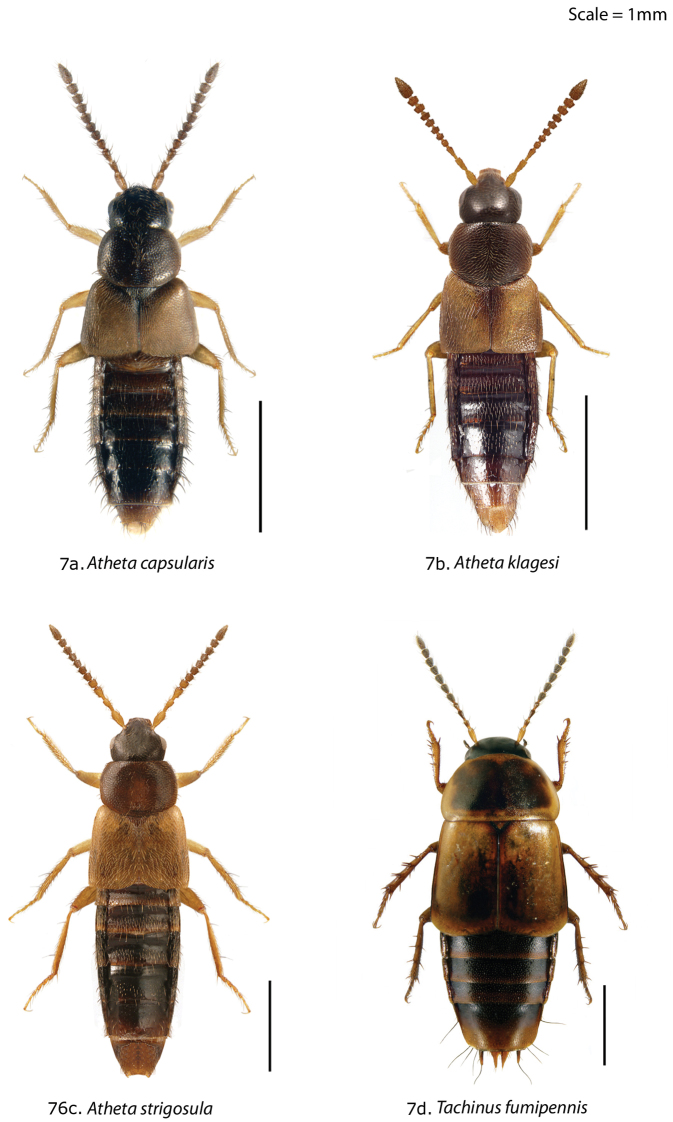
**a–d** Colour images of abundant species: **a** *Atheta capsularis* Klimaszewski **b** *Atheta klagesi* Bernhauer **c** *Atheta strigosula* Casey **d** *Tachinus fumipennis* (Say).

**Figure 8.**
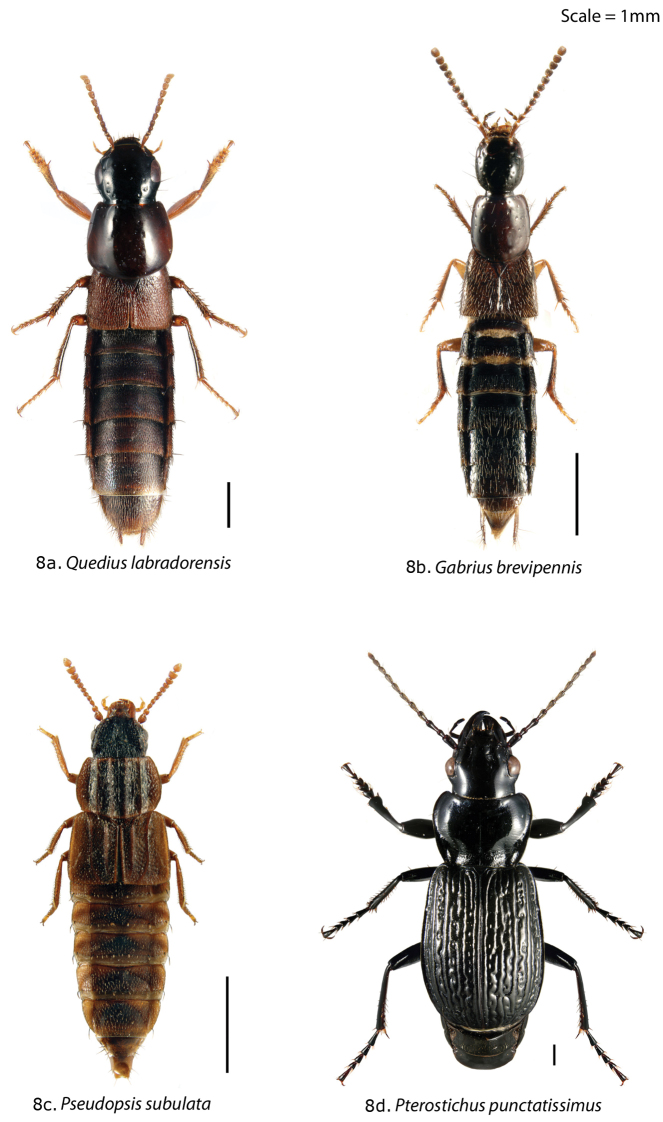
**a–d** Colour images of abundant species: **a** *Quedius labradorensis* Smetana **b** *Gabrius brevipennis* (Horn) **c** *Pseudopsis subulata* Herman **d** *Pterostichus punctatissimus* (Randall).

The sum-of-squares multivariate regression tree divided 11 sites into three nodes which explained 64.5% of the total variance (Table 3, Fig. 6). The initial split explained 46.5% of the variance. This split is attributable to either the treatment difference between uncut and harvested sites or sites that had more than 9 m^3^/ha fine woody debris, both of which offered equal improvement for the multivariate regression tree (improvement by 0.4646). This split was defined by three species of *Atheta* (*Atheta capsularis*, *Atheta klagesi* and *Atheta strigosula*) and *Tachinus fumipennis*/*frigidus* complexcommonly collected in uncut sites and *Gabrius brevipennis*,which was commonly collected in harvested sites.The second split explained an additional 8% of the total variance. This split is attributable to treatment differences between whole-tree and stem-only harvested sites or to sites with more than 112 m^3^/ha of coarse woody debris. Both of these factors provided similar improvement in the overall tree (improvement by 0.2389 and 0.2381 respectively) and could be judged to be nearly equivalent. The second split was defined primarily by *Pseudopsis subulata* commonly collected in sites with reduced volumes of debris.

**Table 3.** Species variance associated with splits in the multivariate regression tree model.

**Species**	**Splits**		**Total Variance Explained**	**Species totals**
	**Harvested vs Control**	**SOH vs WTH**		
*Atheta capsularis*	15.44	0.04	15.48	16.65
*Tachinus fumipennis*	9.63	0.12	9.74	12.45
*Atheta strigulosa*	6.55	0.03	6.58	6.80
*Pseudopsis subulata*	0.68	2.38	3.06	5.82
*Lypoglossa franclemonti*	1.01	0.33	1.33	5.23
*Atheta klagesi*	2.49	0.00	2.49	3.87
*Pterostichus adstrictus*	0.46	1.05	1.52	3.41
*Gabrius brevipennis*	2.02	0.25	2.27	2.62
*Pterostichus coracinus*	0.02	0.06	0.07	2.51
*Atrecus macrocephalus*	0.51	0.12	0.63	2.37
*Ischnosoma fimbriatum*	1.27	0.07	1.34	2.25
*Calathus ingratus*	0.04	1.02	1.06	2.17
*Quedius densiventris*	0.03	0.92	0.94	2.11
*Liogluta aloconotoides*	0.47	0.70	1.17	1.92
*Oxypoda grandipennis*	0.86	0.03	0.89	1.80
*Atheta ventricosa*	0.45	0.00	0.45	1.77
*Mycetoporus americanus*	0.08	0.06	0.14	1.53
*Platynus decentris*	0.29	0.03	0.32	1.41
*Tachyporus nitidulus*	0.33	0.01	0.34	1.27
*Trechus apicalis*	0.13	0.01	0.14	1.27
*Oxypoda pseudolustrica*	0.11	0.13	0.24	1.24
*Tachinus quebecensis*	1.02	0.00	1.02	1.08
*Phloeostiba lapponica*	0.39	0.26	0.66	1.07
Species with < 1% of species totals combined	2.19	2.35	4.54	17.40
Totals	46.47	9.96	56.43	100.00

## Discussion

We were able to detect differences in beetle assemblages among harvested and unharvested plots (Control), and between stem-only (SOH) (i.e. logging residues left on site) and whole-tree (WTH) (i.e. logging residues removed) harvesting treatments. By far the most common species response that we observed was a reduction in abundance in response to removal of the overstory by harvesting, suggesting that at least initially, removal of forest overstory is more important than depletions in the overall volumes of downed deadwood. Of the species with clear responses to harvesting in general, three *Atheta* species and *Atheta fumipennis*/*frigidus* complex had abundances roughly half of those observed in unharvested plots. Similar trends in reduction of abundance caused by harvesting occurred in a yellow birch/balsam fir forest study in Quebec (Klimaszewski et al. 2008). Likewise abundance of several *Atheta*, *Bisnius* and *Gabrius* species was reduced several fold in harvested sites in comparison with uncut forest (Klimaszewski et al. 2008). Similar negative responses to harvesting have been reported for other *Tachinus* species (Pohl et al. 2007).

While we were able to distinguish assemblages in stem-only harvested (SOH) stands from those in whole-tree harvested (WTH) stands, this split was defined primarily on increased catches of *Pseudopsis subulata*. This in itself is interesting as this species was shown to prefer older forests rather than freshly harvested sites in Newfoundland, Canada (McCarthy 1996). Apparently, this species has wider habitat range preferences and can tolerate drier and hotter conditions possibly due to heavy sclerotization of its body integument.

Numerous hypotheses can be advanced to explain these responses, including changes in microclimate (for all species) or loss of potential feeding sites such as decaying mushrooms infested with dipteran larvae (for *Tachinus*). Significant changes in microclimatic conditions occur at the soil surface following removal of harvest residues because the soil becomes more directly exposed to sun radiation and air movement, which cause increased soil temperature and reduce soil moisture (Proe and Dutch 1994, Zabowski et al. 2000, Roberts et al. 2005). Removal of residual slash has also been shown to affect species richness of particular aspects, ground beetles, of the epigaeic fauna (Nitterus 2007). Currently we are not able to positively verify any of these explanations. This is in part because detailed natural history data do not exist for these species beyond extremely broad habitat preferences such as found in leaf litter in forests (Herman 1975). Despite advances in taxonomy, particularly within the Staphylinidae, morphological details published in phylogenetic or taxonomic treatments of small, cryptic beetles like rove beetles have yet to be successfully incorporated into plausible, ecological explanations for species responses. Longer-term monitoring of beetle responses would likely yield clearer species-specific responses, as biodiversity in recently disturbed sites can still reflect a mix of closed canopy and open-habitat species (Buddle et al. 2006, Work et al. 2010).

## Conclusion

Based on 1 year of sampling, we were able to characterize differences in beetle assemblages between clearcut sites (SOH, WTH) and mature stands (Control), as well as differences between clearcut sites where harvest residues had been removed (WTH) or left on site (SOH). The overall assemblage response was largely a recapitulation of the responses of several abundant species. While community-level analysis represents the response of abundantly captured species, we believe that we are likely unable to detect the full extent of the effects of residue removal based on a 1-year experiment. While the MRT allowed us to distinguish different assemblages that were attributable to experimental plots, we were unable to definitively explain assemblage differences on the basis of volumes of either coarse (CWD) or fine woody debris (FWD). This does not necessarily preclude conclusions as to the effect of the silvicultural treatments and the biomass removals, but it does to an extent preclude finer scale mechanistic explanations of changes in particular species. The long-term monitoring studies should allow us to better understand the influence of various levels of postharvest debris removal on biodiversity, nutrient production and circling and eventually tree growth.

## Figures and Tables

**Table 4.** List of rove beetle species (Staphylinidae) reported from the balsam fir/white birch dominated forests (2011) north of Quebec City, organized by subfamily, tribe, subtribe [when available], and species name. All specimens were captured in unbaited pitfall traps. Uncut forest = control (CO); Clearcut with debris (SOH); Clearcut with reduced debris (WTH); Total catch (Σ); Percentage of total catch (%); relative dominance rank (RDR).

**Taxon**	**June**			**July**			**August**			**Catch**		
	**WTH**	**SOH**	**CO**	**WTH**	**SOH**	**CO**	**WTH**	**SOH**	**CO**	**Σ**	**%**	**RDR**
**OMALINAE**	0	1	0	7	2	2	0	0	0	12	0.9	
**Omaliini**	0	1	0	7	2	0	0	0	0	10	0.8	
1. *Omalium rivulare* (Paykull) (*)	0	0	0	0	1	0	0	0	0	1	0.1	20
2. *Phloeostiba lapponica* (Zetterstedt)	0	1	0	7	1	0	0	0	0	9	0.7	15
**Eusphalerini**	0	0	0	0	0	1	0	0	0	1	0.1	
3. *Eusphalerum pothos* (Mannerheim)	0	0	0	0	0	1	0	0	0	1	0.1	20
**Anthophagini**	0	0	0	0	0	1	0	0	0	1	0.1	
4. *Acidota quadrata* (Zetterstedt)	0	0	0	0	0	1	0	0	0	1	0.1	20
**PROTEININAE**	0	0	0	1	1	1	1	0	3	7	0.5	
**Proteinini**	0	0	0	1	1	1	1	0	3	7	0.5	
5. *Megarthrus* sp.	0	0	0	0	0	1	0	0	0	1	0.1	20
6. *Proteinus* sp.	0	0	0	1	1	0	1	0	3	6	0.5	16
**TACHYPORINAE**	6	5	17	13	4	42	2	8	87	184	14.4	
**Tachyporini**	5	2	16	9	3	39	2	6	78	160	12.5	
**7. *Tachinus fumipennis* (Say)**	**0**	**0**	**11**	**2**	**0**	**16**	**0**	**1**	**17**	**47**	**3,7**	**7**
**8. *Tachinus frigidus* Erichson**	**0**	**0**	**5**	**2**	**0**	**20**	**1**	**2**	**46**	**76**	**6,0**	**3**
9. *Tachinus luridus* Erichson	0	1	0	0	0	0	0	0	1	2	0.2	19
10. *Tachinus quebecensis* Robert	0	0	0	0	0	0	0	0	11	11	0.9	14
11. *Tachyporus* sp.	0	0	0	1	1	0	0	0	0	2	0.2	19
12. *Tachyporus nitidulus* (Fabricius)	5	1	0	3	2	1	1	2	0	15	1.2	13
**Mycetoporini**	1	3	1	4	1	3	0	2	9	24	1.9	
13. *Lordithon fungicola* Campbell	0	1	0	0	0	0	0	0	0	1	0.1	20
14. *Mycetoporus consor* LeConte	0	1	0	0	0	0	0	1	0	2	0.2	19
15. *Mycetoporus americanus* Erichson	1	1	1	4	1	3	0	1	9	21	1.6	10
**Bolitobini**	16	6	2	17	18	8	5	4	3	79	6.2	
**16. *Ischnosoma fimbriatum* Campbell**	**13**	**5**	**2**	**16**	**16**	**8**	**4**	**4**	**2**	**70**	**5.5**	**4**
17. *Ischnosoma pictum* (Horn)	3	1	0	1	2	0	1	0	1	9	0.7	15
**ALEOCHARINAE**	34	13	23	14	6	56	53	46	437	682	53.4	
**Aleocharini**	0	0	0	0	0	0	2	2	0	4	0.3	
18. *Aleochara (Xenochara) fumata* Gravenhorst (*)	0	0	0	0	0	0	1	0	0	1	0.1	20
19. *Aleochara (Coprochara) verna* Say (*)	0	0	0	0	0	0	0	1	0	1	0.1	20
20. *Aleochara* sp. n. [undescribed]	0	0	0	0	0	0	1	1	0	2	0.2	19
**Oxypodini**	7	2	2	2	3	3	1	2	19	41	3.2	
21. *Oxypoda frigida* Bernhauer	0	0	0	1	1	0	0	0	1	3	0.2	18
22. *Oxypoda grandipennis* (Casey)	0	0	0	0	0	0	0	1	18	19	1.5	12
23. *Oxypoda lacustris* Casey	0	0	1	0	0	2	0	0	0	3	0.2	18
24. *Oxypoda operta* Sjöberg	1	0	0	0	0	0	0	0	0	1	0.1	20
25. *Oxypoda pseudolacustris* Klimaszewski	6	2	1	1	2	1	1	1	0	15	1.2	13
**Homalotini**	1	0	0	0	0	0	0	0	0	1	0.1	
26. *Leptusa opaca* Casey	1	0	0	0	0	0	0	0	0	1	0.1	20
**Placusini**	0	0	0	0	0	2	0	0	0	2	0.2	
27. *Placusa incompleta* Sjöberg	0	0	0	0	0	2	0	0	0	2	0.2	19
**Athetini**	26	11	21	12	3	51	50	42	417	633	49.5	
**28. *Atheta capsularis* Klimaszewski**	**0**	**0**	**0**	**3**	**0**	**13**	**37**	**31**	**321**	**405**	**31.7**	**1**
**29. *Atheta klagesi* Bernhauer**	**0**	**0**	**9**	**0**	**2**	**28**	**4**	**5**	**15**	**63**	**4.9**	**6**
30. *Atheta regisalmonis* (Lohse)	0	0	0	0	0	0	0	0	1	1	0.1	20
31. *Atheta remulsa* Casey	0	0	0	0	0	0	0	1	1	2	0.2	19
32. *Atheta strigosula* Casey	0	0	0	0	0	2	1	0	64	67	5.2	5
33. *Atheta terranovae* Klimaszewski & Langor	0	0	0	0	0	0	0	0	3	3	0.2	18
34. *Atheta ventricosa* Bernhauer	0	0	11	0	1	3	2	1	1	19	1.5	12
35. *Atheta* sp.	1	0	0	0	0	0	0	0	1	2	0.2	19
36. *Atheta (Microdota)* sp.1	1	0	0	0	0	0	0	0	1	2	0.2	19
37. *Liogluta aloconotoides* Lohse	0	0	0	7	0	5	2	0	6	20	1.6	11
38. *Lypoglossa franclemonti* Hoebeke (*)	22	10	1	2	0	0	3	2	0	40	3.1	8
39. *Mocyta fungi* (Gravenhorst)	0	1	0	0	0	0	0	0	0	1	0.1	20
40. *Mocyta breviuscula* (Mäklin)	0	0	0	0	0	0	0	0	3	3	0.2	18
41. *Seeviersiella globicollis* (Bernhauer)	2	0	0	0	0	0	1	2	0	5	0.4	17
**Geostibini**	0	0	0	0	0	0	0	0	1	1	0.1	
42. *Zyras obliquus* (Casey)	0	0	0	0	0	0	0	0	1	1	0.1	20
STENINAE	1	0	0	1	0	0	0	0	0	2	0.2	
43. *Stenus austini* Casey	1	0	0	1	0	0	0	0	0	2	0.2	19
**PSEUDOPSINAE**	10	6	11	40	16	11	59	10	18	181	14.2	
44. *Pseudopsis subulata* Herman	10	6	11	40	16	11	59	10	18	181	14.2	2
PAEDERINAE	1	2	0	0	0	0	0	0	0	3	0.2	
**Paederini**	1	2	0	0	0	0	0	0	0	3	0.2	
45. *Lathrobium washingtoni* Casey	1	2	0	0	0	0	0	0	0	3	0.2	18
STAPHYLININAE	34	28	5	21	10	12	12	1	5	128	10	
**Xantholinini**	2	10	2	8	7	1	0	0	0	30	2.3	
46. *Atrecus macrocephalus* (Nordman)	2	10	2	8	7	1	0	0	0	30	2.3	9
**Staphylinini**	32	18	3	13	3	11	12	1	5	98	7.7	
**Quediina**	11	3	1	8	1	10	8	0	5	47	3.7	
47. *Quedius densiventris* (Casey)	4	1	0	4	0	2	6	0	3	20	1.6	11
48. *Quedius (Raphirus) fulvicollis* (Stephens)	2	0	0	1	0	1	1	0	0	5	0.4	17
49.*Quedius (Quedius) labradorensis* Smetana	5	1	1	3	1	7	1	0	2	21	1.6	10
50.*Quedius (Quedius) plagiatus* Mannerheim	0	1	0	0	0	0	0	0	0	1	0.1	20
**Philonthina**	21	15	2	5	2	1	4	1	0	51	4	
51. *Bisnius cephalicus* (Casey)	1	1	0	0	0	1	0	0	0	3	0.2	18
**52. *Gabrius brevipennis* (Horn)**	**20**	**14**	**2**	**5**	**1**	**0**	**4**	**1**	**0**	**47**	**3.7**	**7**
53. *Gabrius* sp.	0	0	0	0	1	0	0	0	0	1	0.1	20

**Table 5.** List of ground beetle species (Carabidae) reported from the balsam fir/white birch dominated forests (2011) north of Quebec City, organized by subfamily, tribe, subtribe [when available], and species name. All specimens were captured in unbaited pitfall traps. Uncut forest = control (CO); Clearcut with debris (SOH); Clearcut with reduced debris (WTH); Total catch (Σ); Percentage of total catch (%); relative dominance rank (RDR); abundantly captured species in bold.

**Taxon**	**June**			**July**			**August**			**Catch**		
	**WTH**	**SOH**	**CO**	**WTH**	**SOH**	**CO**	**WTH**	**SOH**	**CO**	**Σ**	**%**	**RDR**
**CARABINAE**	0	0	0	0	0	0	0	1	1	2	0.5	
**Cychrini**	0	0	0	0	0	0	0	1	1	2	0.5	
1. *Sphareoderus nitidicollis* (Guérin-Ménenville)	0	0	0	0	0	0	0	1	1	2	0.5	10
**TRECHINAE**	4	5	2	8	6	6	2	0	0	33	8.5	
**Trechini**	2	2	0	3	4	2	0	0	0	13	3.4	
**2. *Trechus apicalis* (Motschulsky)**	**2**	**1**	**0**	**3**	**4**	**1**	**0**	**0**	**0**	**11**	**2.8**	**6**
3. *Trechus crassiscapus* (Lindroth)	0	1	0	0	0	1	0	0	0	2	0.5	10
**Bembidiini**	2	3	2	5	2	4	2	0	0	20	5.2	
4. *Bembidion grapii* (Gyllenhal)	1	0	0	0	0	0	1	0	0	2	0.5	10
5. *Bembidion wingatei* (Bland)	1	3	2	5	2	4	1	0	0	18	4.7	5
**HARPALINAE**	75	38	69	48	27	44	26	14	11	352	91	
**Pterostichini**	72	34	57	30	25	36	20	12	7	293	75.7	
**6. *Pterostichus adstrictus* (Eschscholtz)**	**58**	**27**	**35**	**21**	**12**	**17**	**12**	**8**	**0**	**190**	**49.1**	**1**
7. *Pterostichus brevicornis* (Kirby)	0	0	1	0	0	0	1	0	0	2	0.5	10
**8. *Pterostichus coracinus* (Newman)**	**10**	**4**	**15**	**9**	**12**	**10**	**5**	**3**	**5**	**73**	**18.9**	**2**
**9. *Pterostichus punctatissimus* (Randall)**	**4**	**3**	**6**	**0**	**1**	**9**	**2**	**1**	**2**	**28**	**7.2**	**4**
**Harpalini**	0	0	0	5	0	0	2	1	0	8	2.1	8
10. *Harpalus laticeps* (LeConte)	0	0	0	0	0	0	0	1	0	1	0.3	11
11. *Harpalus ruficeps* (DeGeer)	0	0	0	3	0	0	1	0	0	4	1	8
12. *Harpalus solitaris* (Dejean)	0	0	0	2	0	0	1	0	0	3	0.8	9
**Platynini**	3	4	12	13	2	8	4	1	4	51	13.2	
13. *Agonum gratiosum* (Mannerheim)	0	0	1	0	0	0	0	0	0	1	0.3	11
14. *Agonum retractum* (LeConte)	0	0	2	0	0	1	0	0	0	3	0.8	9
15. *Calathus adrena* (LeConte)	0	0	1	0	1	0	0	0	0	2	0.5	10
**16. *Calathus ingrathus* (Dejean)**	**2**	**2**	**3**	**13**	**1**	**5**	**4**	**1**	**4**	**35**	**9**	**3**
17. *Platynus decentris* (Say)	1	2	5	0	0	2	0	0	0	10	2.6	7

## Appendix

Colour images of the most abundant rove and ground beetles, and detailed list of specimens examined.
